# Median arcuate ligament syndrome in an old male: a case report with 3-year follow-up

**DOI:** 10.1097/MS9.0000000000001152

**Published:** 2023-08-09

**Authors:** Zein Alabdin Hannouneh, Gieth Alahdab, Amjad Hijazi, Ghaith Harfoush, Chaza Alsayed, Samir Kanaan, Rafah Jamouz

**Affiliations:** a Faculty of Medicine, Al Andalus University for Medical Sciences, Tartus Syrian Arab Republic; b Department of Gastroenterology and Hepatology; c Department of General Surgery; d Department of Medical Imaging and Diagnostic Radiology, Faculty of Medicine, Tishreen University Hospital, Lattakia, Syrian Arab Republic

**Keywords:** case report, celiac artery compression syndrome, Dunbar syndrome, median arcuate ligament syndrome, vascular compression syndrome

## Abstract

**Introduction and importance:**

Median arcuate ligament syndrome (MALS) or Dunbar syndrome is a rare compression syndrome that poses a challenge to many clinicians due to its ambiguous symptoms. It is predominantly common in females in their 30s to 50s.

**Case presentation:**

A 74-year-old male presented with generalized chronic postprandial abdominal pain, anorexia, and weight loss of 6 kg for the past 2 months. Physical examination, abdominal ultrasound, endoscopy, and colonoscopy were all unremarkable. His laboratory workup and tumor marker tests were within normal ranges. Finally, a multi-slice computed tomography (MSCT), an advanced computed tomography with multiple detectors resulting in faster and higher resolution imaging, outlined external compression on the celiac artery (CA) by the median arcuate ligament (MAL). The release of the CA from the MAL was done laparoscopically. Symptoms improved significantly postoperatively. During the follow-up period of 3 years, the patient did not regain his lost weight but had no other complaints.

**Clinical discussion:**

Due to its vague manifestations, MALS is diagnosed only after extensive evaluation and exclusion. This challenging diagnosis outlines the need for refined diagnostic guidelines. An MSCT plays a crucial role in confirming the diagnosis. Currently, more physicians prefer laparoscopic release of the MAL compared to an open approach.

**Conclusion:**

Despite MALS predominance in females, the diagnosis of MALS should be considered in males with postprandial abdominal pain and unexplained weight loss. An MSCT, along with other imaging modalities, can provide a comprehensive view of celiac compression. Laparoscopic decompression of the CA is an ideal treatment option.

## Introduction

Highlights

- Median arcuate ligament syndrome (MALS) is an uncommon syndrome that typically presents with postprandial epigastric pain and unexplained weight loss.
- MALS is a challenging diagnosis of exclusion, and further studies are needed to reform diagnostic guidelines to provide patient relief as early as possible. A multi-slice computed tomography (MSCT) plays an important role in confirming the diagnosis.
- Clinicians need to keep in mind MALS in patients with postprandial epigastric pain, unintentional weight loss, and loss of appetite, despite an unclassical demographic.
- Compared to an open approach, laparoscopic decompression of the celiac artery is advantageous, especially in centers with experience.

The median arcuate ligament (MAL) is a continuation of the posterior diaphragm that wraps over the aorta and passes superior to the origin of the celiac artery^1^. In rare cases, the ligament can lead to significant compression of the celiac artery (CA). Median arcuate ligament syndrome (MALS), also known as Dunbar syndrome or celiac artery compression syndrome, is a rare vascular compression syndrome that poses a challenge to many clinicians due to its nonspecific symptoms, including exercise-induced or postprandial abdominal pain, nausea, vomiting, and weight loss, and lack of consensus on a diagnostic criterion^1^. The etiology behind these symptoms is not fully understood, and management revolves around a multitude of methods that includes MAL release with or without celiac gangliectomy and angioplasty through open laparotomy, retroperitoneal endoscopy, laparoscopy, or robot-assisted surgery^1^. We present a case of a 74-year-old male with MALS that caused abdominal pain and weight loss. This case report has been reported in line with the SCARE (Surgical CAse REport) Criteria^2^.

## Case presentation

A Syrian male in his 70s presented to the gastroenterology department with chronic epigastric pain and loss of appetite as his main complaints. His generalized abdominal pain was worse with food ingestion, not associated with the position, and persistent for the past 2 months despite antacid use. He unintentionally lost 6 kg of weight since the onset of his symptoms but reported no nausea, vomiting, bloating, or change in defecation habits. He endured a significant loss of appetite a few weeks before his presentation. His medical and drug histories were unremarkable, aside from over-the-counter medications. He denied smoking or drinking alcohol.

Physical examination was unremarkable. His vital signs included a body temperature of 37°C, 95% oxygen saturation, 110/70 mmHg arterial blood pressure, and a heart rate of 70 beats per minute. His initial laboratory workup, including a complete blood count with differential kidney functions, liver functions, thyroid function tests, electrolytes, and serum and urinary amylase, was within normal range. Tumor markers CA (carbohydrate antigen) 19-9, CEA (carcinoembryonic antigen), and PSA (prostate-specific antigen) were 8.43, 1.06, and 6.6 µg/l, respectively.

An abdominal ultrasound revealed no abnormal findings. The patient was then referred for an upper gastrointestinal endoscopy which uncovered an uncomplicated small hiatal hernia with linear hypertrophic gastropathy in the body and antrum of the stomach. A biopsy of the duodenum revealed chronic duodenitis without villous atrophy (Marsh score 0). Additionally, a lower colonoscopy incidentally found internal hemorrhoids (grade I) with papilloma through retroflexion of the scope.

**Figure 1:**
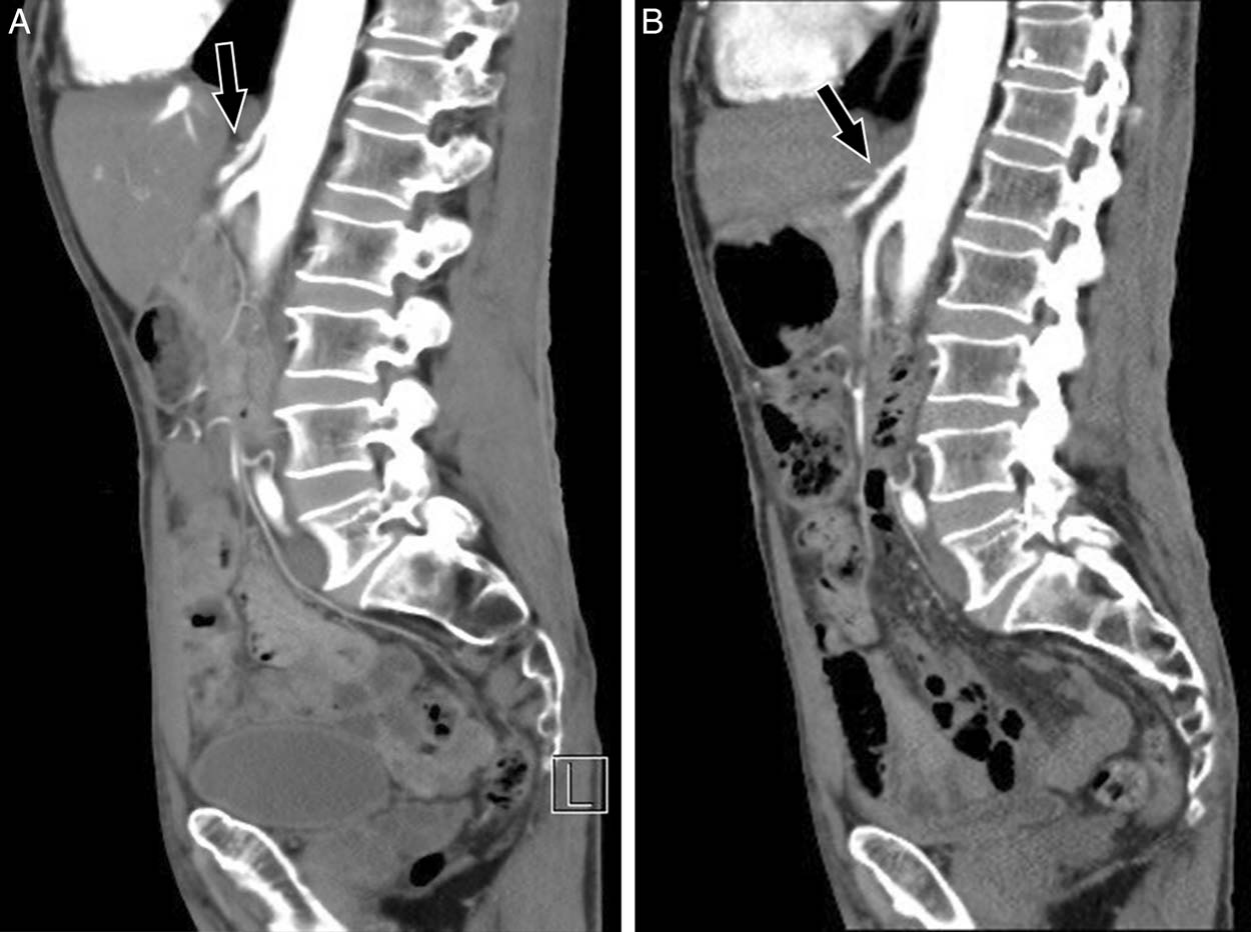
(A) Presurgical abdominal multi-slice computed tomography (MSCT) with contrast (sagittal view): focal mild compression of the celiac trunk is present. The celiac artery is slightly driven down (arrow) due to extrinsic compression by a thickened median arcuate ligament >7 mm (normal 4 mm). (B) Postsurgical abdominal MSCT with contrast (sagittal view): after laparoscopic release of the median arcuate ligament, the celiac trunk is shown in the normal position (arrow) with no observed stenosis.

Despite extensive investigations into the cause of his abdominal pain, no underlying etiology could be recognized. The decision was made to perform a multi-slice computed tomography (MSCT) with contrast. The MSCT revealed mild narrowing near the origin of the CA along with a thickened MAL (>7 mm) (Fig. 1A). These findings confirmed the diagnosis of MALS.

**Figure 2:**
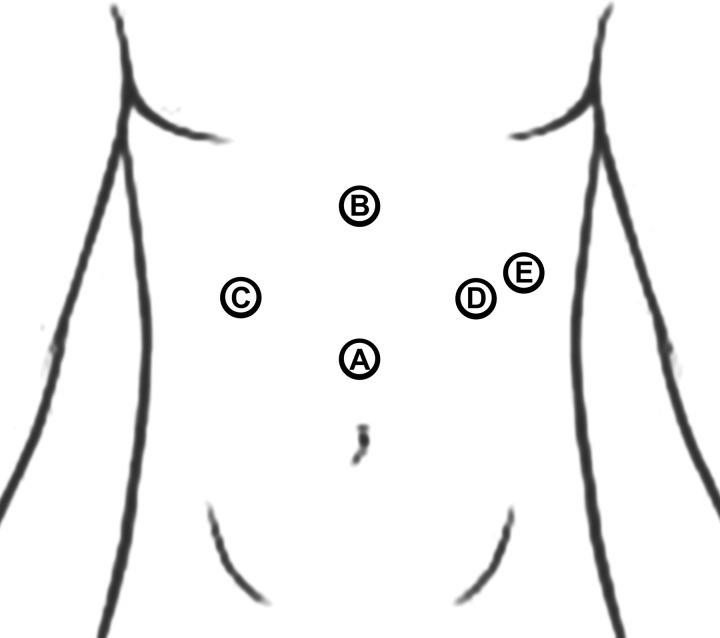
Five laparoscopic ports were used in the demonstrated positions. (A) Camera probe, (B) liver retractor, (C), (D), and (E) surgeon’s operating instruments.

**Figure 3:**
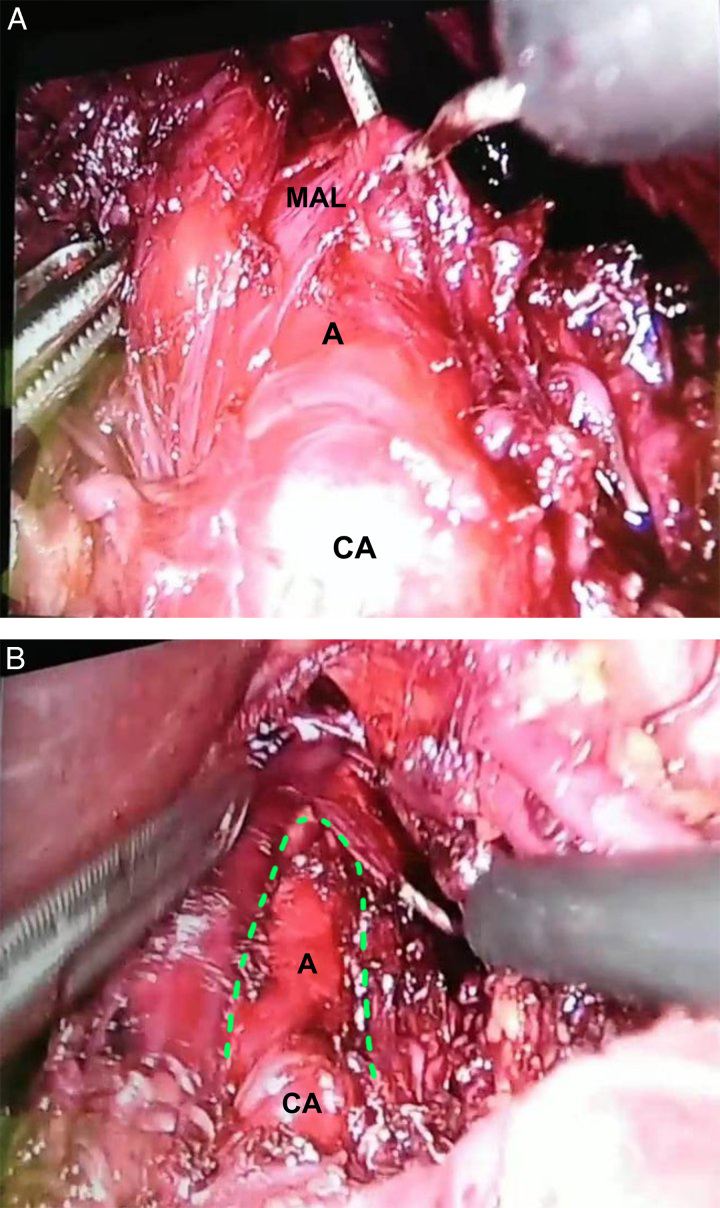
(A) Laparoscopic isolation of the celiac artery (CA) as it branches out of the aorta (A) from the compressing median arcuate ligament (MAL). (B) View of the decompressed celiac artery (CA) and the abdominal aorta (A) after cutting the median arcuate ligament (dotted line).

A laparoscopic surgical procedure was scheduled to relieve extrinsic pressure from the CA by cutting the MAL. The surgery was conducted by a senior surgeon, and five laparoscopic ports were used (Fig. 2) after general anesthesia to access the celiac trunk through the lesser omentum. After isolating the CA (Fig. 3A), the MAL was cut by a monopolar electrocautery probe, which resolved the stenosis (Fig. 3B). The surgery was concluded after the placement of a Penrose drainage tube, which was later removed after 24 h. The patient was started on clear oral liquids, which were gradually increased, reaching a free oral diet on day 3 post-operation. The patient had an uneventful hospital stay and recovered without complications.

Another MSCT was conducted after the procedure. It confirmed the successful release of extrinsic pressure over the patent CA (Fig. 1B). The patient reported significant symptom relief postoperatively and was discharged on day 4 post-operation. His appetite recovered, though he did not regain his lost weight during the follow-up period of 3 years.

## Discussion

MALS, which is also known as Dunbar or celiac axis/artery compression syndrome, is an uncommon disorder characterized by compression of the celiac trunk by the MAL, leading to decreased blood demand and eventually foregut ischemia. The disorder was first described by Harjola in 1968^3^. Only one case has been described within Syria^4^. With an incidence of 2 per 100 000^5^, MALS is most common in 30–50-year-old females (4:1 ratio) with thin stature^6^. Many cases of MAL compression are found incidentally in the general population, and the majority remain asymptomatic. In a review of 400 asymptomatic patients, 7.3% demonstrated significant (>50% luminal narrowing and a pressure gradient ≥10 mmHg) celiac axis stenosis^7^. In symptomatic patients, symptoms typically include postprandial abdominal pain and unexplained weight loss. Other vague symptoms, such as nausea, vomiting, and diarrhea, can occur^8^. Moreover, a physical examination may reveal nonspecific abdominal tenderness and bruits, exaggerated on expiration^6^.

Because of its imitation of many abdominal disorders, MALS is usually a challenging diagnosis of exclusion. As a result, patients undergo extensive assessment with many inconclusive tests before arriving at a diagnosis. Duplex ultrasound is a common initial screening tool. Its preference relies on being noninvasive, inexpensive, and helpful in detecting at least 50% and at least 70% celiac artery stenosis through peak systolic velocity^9^. However, it is dependent on the operator’s experience. Furthermore, the patient body habitus and overlying bowel gas can limit its accuracy. Additional modalities, such as computed tomography angiography and magnetic resonance angiography, can be used to visualize CA compression in a three-dimensional view and confirm MALS^8^.

The primary objective of MALS treatment is to relieve the overlying pressure from the celiac axis. The conventional method of treatment is through an open laparotomy that includes decompression alone, decompression with celiac dilatation, or decompression with reconstruction. Laparoscopic MAL release is becoming the modern standard for treating MALS due to its several advantages (less morbidity, less blood loss, less postoperative pain, fewer adhesions, and better cosmetic results)^10^. However, the rate of conversion to open surgery is relatively high (9.1%) and may be attributed to the learning curve associated with this technique^11^. Intraoperative ultrasonography is usually advised to assess CA flow after decompression. However, its use might be unnecessary because of reports outlining the resolution of symptoms both with and without ultrasonography^12,13^. Moreover, postoperative percutaneous transluminal angioplasty (PTA) may be used in combination with laparoscopy and has shown good outcomes in patients with recurrent artery stenosis after MAL decompression^6^.

In our case, the patient was a 74-year-old male, which is outside the classical demographic of younger women with thin body habitus. The patient’s old age and presenting symptoms of chronic postprandial pain, weight loss, and anorexia prompted the search for gastrointestinal (GI) tumors and other causes of obstruction. Peptic ulcer disease, celiac disease, inflammatory bowel diseases, and mesenteric ischemia were also suspected. After indeterminate, normal ultrasound findings and laboratory workup, upper endoscopy and colonoscopy excluded diseases, tumors, or obstruction of the GI tract. A duplex ultrasound could not be obtained due to the lack of an experienced practitioner at the time. An MSCT with contrast confirmed the diagnosis of MALS and excluded atherosclerosis. Laparoscopy was the preferred approach to decompress the CA in our patient. Intraoperative ultrasonography and PTA were not used during the procedure. Symptom relief occurred immediately after surgery. During the 3-year follow-up period, the patient continued to have no symptoms but did not regain his lost weight.

## Conclusion

We present a case of MALS in an unclassical demographic of a 74-year-old male. After an indeterminate extensive evaluation, MALS was diagnosed with an MSCT, and laparoscopy was performed accordingly. The patient did not regain his lost weight but had significant symptom relief postoperatively. This case outlines the importance of keeping MALS as a differential diagnosis in cases of vague gastrointestinal symptoms and the need for better diagnostic guidelines due to the challenging presentation of this syndrome.

## Ethical approval

Given the nature of the article, a case report, no ethical approval was required.

## Consent

Written informed consent was obtained from the patient for the publication of this case report and accompanying images. A copy of the written consent is available for review by the Editor-in-Chief of this journal on request.

## Sources of funding

None.

## Author contribution

Z.A.H., G.A., A.H., and G.H.: writing – original draft, review, and editing; C.A.: supervision, final review and editing, and patient’s gastroenterology care and follow-up; S.K.: supervision, review and editing, and patient’s surgical care; R.J.: supervision, review, and editing, and performed patient’s imaging. All authors read and approved the final manuscript.

## Conflicts of interest disclosure

The authors declare that they have no conflicts of interest.

## Research registration unique identifying number (UIN)

None.

## Provenance and peer review.

Not commissioned, externally peer-reviewed.

## Data availability statement

None.

## Guarantor

Chaza Alsayed, MD.
